# Ixazomib with cyclophosphamide and dexamethasone in relapsed or refractory myeloma: MUKeight phase II randomised controlled trial results

**DOI:** 10.1038/s41408-022-00626-4

**Published:** 2022-04-01

**Authors:** Holger W. Auner, Sarah R. Brown, Katrina Walker, Jessica Kendall, Bryony Dawkins, David Meads, Gareth J. Morgan, Martin F. Kaiser, Mark Cook, Sadie Roberts, Christopher Parrish, Gordon Cook

**Affiliations:** 1 Department of Immunology and Inflammation and The Hugh and Josseline Langmuir Centre for Myeloma Research, Imperial College London, London, UK; 2 Clinical Trials Research Unit, Leeds Institute of Clinical Trials Research, University of Leeds, Leeds, UK; 3 Academic Unit of Health Economics, Leeds Institute of Health Sciences, University of Leeds, Leeds, UK; 4 Perlmutter Cancer Center, NYU Langone Health, New York, NY USA; 5 The Institute of Cancer Research, London, UK; 6 The Royal Marsden Hospital NHS Foundation Trust, London, UK; 7 Centre for Clinical Haematology, Queen Elizabeth Hospital, Birmingham, UK; 8 Department of Clinical Haematology, St. James’s University Hospital, Leeds, UK

**Keywords:** Drug development, Cancer therapy, Cancer therapy

## Abstract

The all-oral combination of ixazomib, cyclophosphamide, and dexamethasone (ICD) is well tolerated and effective in newly diagnosed and relapsed multiple myeloma (MM). We carried out MUKeight, a randomised, controlled, open, parallel group, multi-centre phase II trial in patients with relapsed MM after prior treatment with thalidomide, lenalidomide, and a proteasome inhibitor (ISRCTN58227268), with the primary objective to test whether ICD has improved clinical activity compared to cyclophosphamide and dexamethasone (CD) in terms of progression-free survival (PFS). Between January 2016 and December 2018, 112 participants were randomised between ICD (*n* = 58) and CD (*n* = 54) in 33 UK centres. Patients had a median age of 70 years and had received a median of four prior lines of therapy. 74% were classed as frail. Median PFS in the ICD arm was 5.6 months, compared to 6.7 months with CD (hazard ratio (HR) = 1.21, 80% CI 0.9–1.6, *p* = 0.3634). Response rates and overall survival were not significantly different between ICD and CD. Dose modifications or omissions, and serious adverse events (SAEs), occurred more often in the ICD arm. In summary, the addition of ixazomib to cyclophosphamide and dexamethasone did not improve outcomes in the comparatively frail patients enroled in the MUKeight trial.

## Introduction

In the past 2 decades, treatment options for multiple myeloma (MM) have increased dramatically. The emergence of several new drug classes, and their combination with each other and conventional agents in a vast array of regimens, have altered the therapeutic landscape extensively. Treatment options for relapsed/refractory MM (RRMM) are particularly plentiful and continue to expand with the development of immunotherapy approaches that include chimeric antigen receptor (CAR)-T cells and bi-specific T-cell engagers [1, 2]. While these developments hold great promise, many of the new treatment approaches will, for the foreseeable future, be inaccessible to large numbers of MM patients globally as they are often costly and complex to deliver. Even in healthcare systems with the capacities to deliver novel therapeutic strategies, the SARS-CoV2 pandemic has highlighted the need for easy-to-adminster regimens that require limited contact of patients with healthcare providers. Such regimens are also beneficial for patients with impaired access to healthcare facilities for reasons such as geographical remoteness, and for old or frail patients, particularly in an advanced disease setting.

Proteasome inhibitors (PIs) have been instrumental in shaping myeloma therapy over the past 2 decades and remain a cornerstone of treatment regimens in newly diagnosed MM (NDMM) and RRMM [1, 2]. PIs have proven highly efficacious in combination with drugs with novel mechanisms of action such as the so-called immunomodulatory drugs and monoclonal antibodies. However, PIs have also shown excellent anti-myeloma activity in combination with alkylating agents. The combination of bortezomib or carfilzomib with dexamethasone and melphalan (VMP, KMP) leads to high response rates in NDMM and RRMM [3, 4]. Cyclophosphamide, when given in combination with dexamethasone and bortezomib or carfilzomib is also effective in NDMM and RRMM [5–12]. Both PIs, however, require parenteral administration, and while peripheral neuropathy remains a prominent side effect of bortezomib, cardiovascular adverse events are of concern in regimens containing carfilzomib.

Ixazomib is an oral small molecule inhibitor of the proteasome that was approved by the US Food and Drug Administration and European Medicines Agency in combination with lenalidomide and dexamethasone in patients with at least one prior line of therapy, based on the phase 3 TOURMALINE-MM1 trial [13]. The combination of ixazomib with lenalidomide has also been shown to be well tolerated and effective in NDMM [14, 15]. Clinical trials investigating ixazomib with thalidomide or pomalidomide have also yielded encouraging results in upfront and relapsed therapy settings [16–18]. However, while these regimens are all oral, the so-called immunomodulatory drugs may not be well suited for subsets of patients, such as those with underlying severe renal impairment, increased risk of thromboembolic complications, or those progressing on an immunomodulatory agent. Based on the results of other PIs in combination with alkylating agents and steroids, a regimen of ixazomib with dexamethasone and cyclophosphamide is a potentially attractive all-oral three-drug approach, a notion that is supported by observations in NDMM and RRMM patients [19–21]. We therefore conducted a randomised phase II trial of ixazomib combined with cyclophosphamide and dexamethasone (ICD), compared to cyclophosphamide plus dexamethasone (CD), in RRMM patients who have relapsed after treatment with thalidomide, lenalidomide and a PI, to determine whether the addition of ixazomib offers increased progression-free survival.

## Methods

The full trial protocol, including eligibility criteria and sample size, has been previously published [22]. Key eligibility criteria included prior treatment with (but not refractoriness to) thalidomide, lenalidomide and a proteasome inhibitor and ECOG performance status ≤2. The trial received national research ethics approval from the NHS National Research Ethics Service Liverpool East (REC Number: 15/NW/0416) and is registered on the International Standard Randomised Controlled Trial Number register (ISRCTN58227268). Participants were randomised (1:1, ICD:CD) from 33 UK centres. Randomisation was performed centrally by the University of Leeds Clinical Trials Research Unit (CTRU), using minimisation with a random element to balance for age (<60 vs. 60–69 vs. ≥70), number of prior lines of therapy (>3 vs. ≥3) and β2 microglobulin (<3.5 mg/L vs. 3.5–5.5 mg/L vs. ≥5.5 mg/L). Upon centrally confirmed disease progression eligible participants in the CD arm were permitted to crossover to treatment with ICD (protocol amendment 23rd May 2017). Written informed consent was collected for all participants.

Ixazomib was prescribed 4 mg orally on days 1, 8 and 15 of a 28-day treatment cycle, with cyclophosphamide 500 mg orally on days 1, 8 and 15 and dexamethasone 40 mg orally on days 1–4 and 8–12. For older/less fit participants (as determined by the principal investigator), the starting dose of dexamethasone could be reduced to 20 mg, days 1–4, and 12–15. Participants received treatment until disease progression, intolerance or participant withdrawal. For dose reduction schedule, see supplementary material Fig. S1.

The primary endpoint was progression-free survival (PFS) defined as time from randomisation to first evidence of disease progression or death. Secondary endpoints included response to treatment ((≥partial response (PR)), maximum response, time to maximum response, duration of response, overall survival (OS), treatment compliance, safety and toxicity, quality of life and cost-effectiveness (not reported here). All endpoints with the exception of OS were assessed prior to treatment crossover. Exploratory endpoints post-crossover included PFS, response endpoints, treatment compliance, safety and toxicity.

Responses were defined according to IMWG guidelines [23]. Safety & toxicity data were graded using NCI CTCAE v4.0. Bone marrow myeloma tumour cells were purified (>95%) in a central laboratory using CD138 immunomagnetic selection (Miltenyi Biotech, Bergisch Gladbach, Germany). Recurrent tumour immunoglobulin locus translocations t(4;14) and t(14;16)/t(14;20) were assessed using qRT-PCR (Life Technologies/Thermo Fisher Scientific, Darford, UK) and copy number aberrations del (1p), gain (1q) and del (17p) using multiplex ligation-dependent probe amplification (MLPA; probemix P425; MRC-Holland, Amsterdam, The Netherlands), as previously described [24].

A total sample size of 250 patients was required to detect an improvement in median PFS from 6 to 9 months with the addition of ixazomib to CD, based on data from previous studies [25–30], corresponding to a hazard ratio of 0.67. With 80% power and overall 2-sided 5% significance level, a total of 198 PFS events were required to be observed. One formal interim efficacy analysis was planned when 70% of PFS events had occurred. Owing to slower than anticipated recruitment, and in discussion with the independent Data Monitoring and Ethics Committee (DMEC) and Trial Steering Committee (TSC) a revised sample size of 140 patients was planned, based on an inflated two-sided 20% significance level and no interim analysis.

All analyses were pre-planned, unless specified, and performed as two-sided tests. The analysis population was defined as all participants who received at least one dose of any trial treatment. PFS, time to maximum response, duration of response and OS were analysed using Kaplan–Meier curves, a log-rank test and Cox’s proportional hazards model, adjusting for minimisation factors. No adjustment for crossover was made for OS analyses. Response was analysed using logistic regression, adjusting for minimisation factors. Safety, toxicity and treatment compliance were summarised descriptively. All crossover endpoints were summarised descriptively from time of treatment crossover, for those patients in the CD arm who received further ICD treatment. On the basis of the revised sample size, 80%CIs were calculated for all endpoints. Analysis was performed in SAS v9.4, by JK, KW, and SRB at CTRU. All authors had access to clinical trial results.

Data sharing statement: Any requests for individual participant data will be reviewed by the trial management group in the first instance. Only requests that have a methodologically sound proposal and whose proposed use of the data has been approved by the independent trial steering committee will be considered. Proposals should be directed to the corresponding author in the first instance; to gain access, data requestors will need to sign a data access agreement. The study protocol is publicly available [22].

## Results

**Fig. 1:**
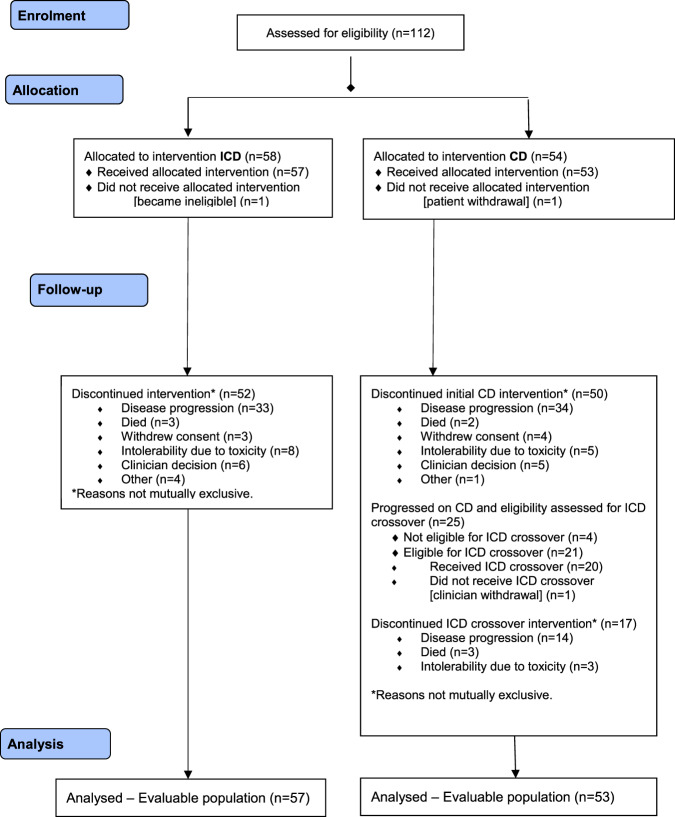
Clicnial Trial Consort flow diagram.

Between January 2016 and December 2018, 112 participants from 33 UK centres were randomised between ICD (*n* = 58) and CD (*n* = 54), closing early at the recommendation of the DMEC due to continued slow recruitment. Data download for final analysis took place on 4th November 2019, with median follow-up of 10.7 months. At the time of download 8 patients were still receiving treatment. The analysis population consisted of 110 participants (ICD: 57, CD: 53), as two participants received no treatment (1 withdrew before treatment, 1 became ineligible). Figure 1 summarises the flow of participants through the trial. Of the 34 patients who discontinued CD due to disease progression, 7 discontinued prior to the protocol amendment for treatment crossover, 1 patient received no further treatment, and 1 patient was not assessed for eligibility. A total of 25 CD patients were therefore assessed for crossover to ICD, of whom 21 were eligible and 20 received ICD. Baseline characteristics were generally well balanced between the arms (Table 1), with a median age of 70 years (range 46–82). In the entire study population, 73.6% (81/112) participants had a Charlson Comorbidity Index score of 0–2. More participants in the ICD arm had ECOG PS 1 or 2 (78.9% vs. 66.0%), and were classed as frail (80.7% vs. 66.0%) as determined by the modified iMWG frailty score [31]. There was a median of 4 (range 1–5+) prior lines of therapy, and median time from diagnosis to trial entry was 6.8 years (range 1.8–21.0). Complete genetics data were available for 48 patients, and partial data for 19.

**Table 1:** Minimisation factors and baseline characteristics.

		ICD (*n* = 57) *N* (%)	CD (*n* = 53) *N* (%)	Total (*n* = 110) *N* (%)
Minimisation factors				
Age at randomisation	<60	10 (17.5)	10 (18.9)	20 (18.2)
	60–69	18 (31.6)	15 (28.3)	33 (30.0)
	70+	29 (50.9)	28 (52.8)	57 (51.8)
Number of prior lines of therapy	>3	45 (78.9)	44 (83.0)	89 (80.9)
	≤3	12 (21.1)	9 (17.0)	21 (19.1)
β2 microglobulin	< 3.5 mg/L	22 (38.6)	22 (41.5)	44 (40.0)
	3.5–5.5 mg/L	20 (35.1)	16 (30.2)	36 (32.7)
	≥5.5 mg/L	15 (26.3)	15 (28.3)	30 (27.3)
Baseline characteristics				
Age	Median (range)	70.0 (49.0, 82.0)	70.0 (46.0, 82.0)	70.0 (46.0, 82.0)
Gender	Male	33 (57.9)	30 (56.6)	63 (57.3)
	Female	24 (42.1)	23 (43.4)	47 (42.7)
Prior lines of therapy	Median (range)	5 (1, 5 + )	4 (1, 5 + )	4 (1, 5 + )
	>4	29 (50.8)	24 (46.2)	53 (48.2)
	≤4	28 (49.2)	29 (53.8)	57 (51.8)
Prior Therapies	Bortezomib	56 (98.2)	51 (96.2)	107 (97.3)
	Lenalidomide	57 (100.0)	53 (100.0)	110 (100.0)
	Thalidomide	55 (96.5)	52 (98.1)	107 (97.3)
	Anti-CD38	11 (19.3)	9 (17.0)	20 (18.2)
	Prior ASCT	30 (52.6)	29 (55.8)	59 (53.6)
Time since diagnosis (years)	Median (range)	7.3 (1.8, 21.0)	6.7 (2.1, 20.5)	6.8 (1.8, 21.0)
ECOG performance status	0	12 (21.1)	18 (34.0)	30 (27.3)
	1	40 (70.2)	28 (52.8)	68 (61.8)
	2	5 (8.8)	7 (13.2)	12 (10.9)
Charlson Comorbidity Index score	0-2	41 (71.9)	40 (75.5)	81 (73.6)
	>2	16 (28.1)	13 (24.5)	29 (26.4)
Frailty score^a^	Frail	46 (80.7)	35 (66.0)	81 (73.6)
	Non-frail	11 (19.3)	18 (34.0)	29 (26.4)
	Median (range)	2 (1-5)	2 (1–4)	2 (1-5)
ISS at baseline	I	15 (26.3)	14 (26.4)	29 (26.4)
	II	23 (40.4)	23 (43.4)	46 (41.8)
	III	17 (29.8)	16 (30.2)	33 (30.0)
	Not available/missing	2 (3.6)	0	2 (1.8)
Genetics data at baseline	Full	27 (47.4)	21 (39.6)	48 (43.6)
	Partial	7 (12.3)	12 (22.6)	19 (17.3)
	Not available	23 (40.4)	20 (37.7)	43 (39.1)
High risk lesions (*n* = 67)	Del1p	3 (8.8)	6 (18.2)	9 (13.4)
	Gain1q	16 (47.1)	20 (60.6)	36 (53.7)
	Del17p	8 (23.5)	5 (15.2)	13 (19.4)
High risk lesions (*n* = 48)	t(4;14)	2 (7.4)	0 (0)	2 (4.2)
	t(14;16) / t(14,20)	2 (7.4)	2 (9.5)	4 (8.3)

^a^Frailty was determined using age, Charlson Comorbidity Index and ECOG performance status (Facon et al., 2020).

### Response to treatment, progression-free, and overall survival

Response rates were similar between arms (Table 2), with 24/57 participants (42.1%, 80% CI 33.2–51.5) in the ICD arm, and 21/53 (39.6%, 80% CI 30.5–49.4) in the CD arm, achieving at least PR as their maximum response. Seven patients (3 on ICD, 4 on CD arm) stopped treatment after cycle 1 and did not complete a response assessment. In logistic regression analysis the odds ratio for the overall response rate (ORR; ≥ PR, ICD vs. CD) was 1.1 (80% CI 0.66–1.84, *p* = 0.8015). No minimisation factors were significantly associated with ORR. Median time to maximum response was 2.1 months for ICD and 1.9 months for CD (unadjusted HR 1.14, 80% CI 0.86–1.49). Median duration of response was 6.3 months for ICD and 10.8 months for CD (unadjusted HR 1.23, 80% CI 0.79–1.93). Median time to progression was 5.8 months for ICD and 6.7 months for CD (HR = 1.13, 80% CI 0.86–1.50, *p* = 0.5634).

**Table 2:** Maximum response.

Maximum response	ICD (*n* = 57)	CD (*n* = 53)	Total (*n* = 110)
CR	1 (1.8%)	1 (1.9%)	2 (1.8%)
VGPR	8 (14.0%)	5 (9.4%)	13 (11.8%)
PR	15 (26.3%)	15 (28.3%)	30 (27.3%)
MR	9 (15.8%)	6 (11.3%)	15 (13.6%)
SD or NC	14 (24.6%)	18 (34.0%)	32 (29.1%)
PD	7 (12.3%)	4 (7.5%)	11 (10.0%)
No maximum response	3 (5.3%)	4 (7.5%)	7 (6.4%)

**Fig. 2:**
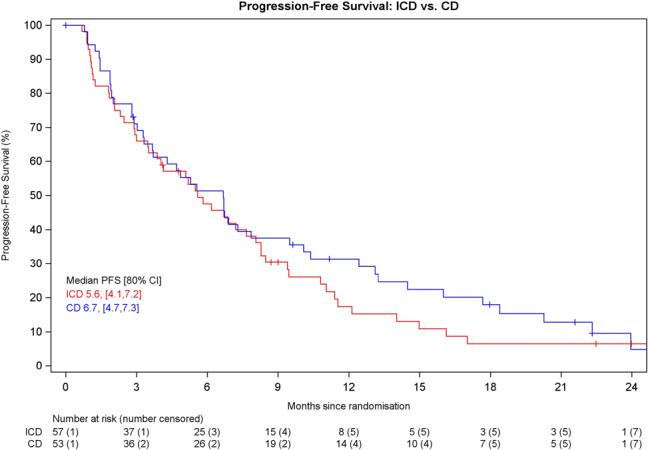
Progression-free survival.

Median PFS in the ICD arm was 5.6 months (80% CI 4.1–7.2), compared to 6.7 months (80% CI 4.7–7.3) with CD (hazard ratio (HR) = 1.21, 80% CI 0.9–1.6, *p* = 0.3634) (Fig. 2). Proportional hazards assumptions were violated with the inclusion of age as a prognostic factor therefore this variable was removed from the model and test assumptions were satisfied. No prognostic factors were found to be significantly associated with PFS.

**Fig. 3:**
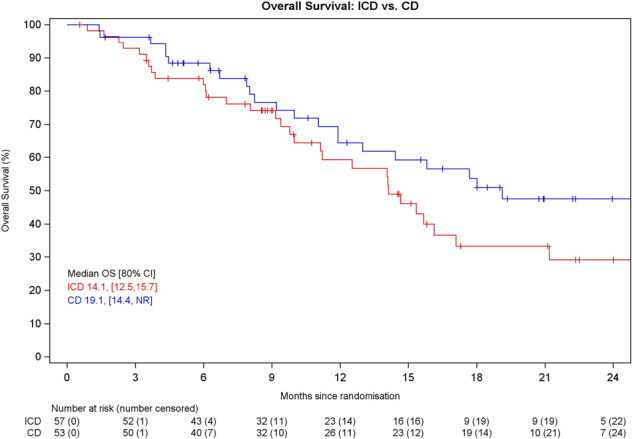
Overall survival.

Overall survival was not significantly different between the arms, with median overall survival 14.1 months for ICD vs. 19.1 months for CD (HR = 1.52, 80% CI 1.06–2.18, *p* = 0.1346) (Fig. 3). At the time of analysis 54 patients had died (31 on ICD, 23 on CD arm).

**Fig. 4:**
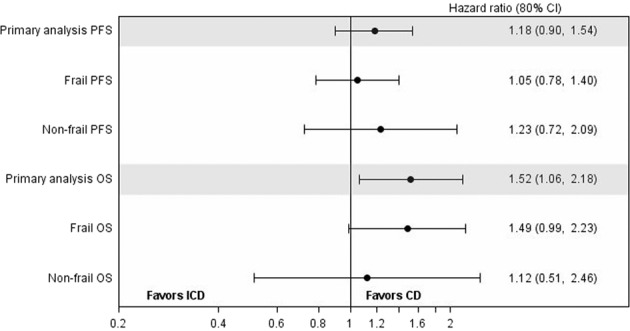
Forest plots of progression-free and overall survival by frailty group.

Exploratory analyses were performed to assess the impact of frailty on PFS and OS, however no significant differences between frailty groups were identified. 96/110 (87.3%) participants had a progression event (non-frail: 25/29, 86.2%; frail: 71/81, 87.6%), and 54/110 (49.1%) participants died (non-frail: 13/29, 44.8%; frail: 41/81, 50.6%). Median survival estimates by treatment and frailty group are shown in Table 3, and forest plots of PFS and OS in Fig. 4. No significant interactions were observed.

**Table 3:** Median survival estimates (months) by treatment and frailty group.

		ICD Median (80% CI)	CD Median (80% CI)
PFS	Non-frail	5.1 (3.5–5.6)	7.0 (4.7–16.0)
	Frail	6.7 (4.1–8.0)	5.6 (3.7–6.9)
OS	Non-frail	15.3 (12.5–3.08)	*Not estimated*
	Frail	14.1 (11.1–15.7)	18.0 (13.0-*not estimated)*

### Treatment compliance and toxicity

The median number of cycles received was 4 in both arms (ICD range 1–29, CD range 1–24). The majority of patients stopped treatment due to disease progression (Supplementary Table 1), with 7 patients (13.5%) in the ICD arm and 5 (10.0%) in the CD arm discontinuing solely due to toxicity. Mean doses delivered of dexamethasone and cyclophosphamide were similar between the arms (Supplementary Table 2), and mean Ixazomib dose received was 3.7 mg (SD 0.69). Dose modifications to any treatment were reported for 41 participants in the ICD arm (71.9%) and 29 in the CD arm (54.7%). Similar numbers of participants experienced dose reductions (ICD 23/57, 40.4%; CD 25/53, 47.2%), whereas more ICD participants experienced dose omissions (ICD 35/57, 61.4%; CD 14/53, 26.4%).

Higher rates of grade ≥3 thrombocytopenia, neutropenia, diarrhoea, sensory neuropathy, fatigue and infection were seen in the ICD arm, compared to the CD arm (Table 4). More participants in the CD arm experienced hypotension and hyperglycaemia.

**Table 4:** Adverse reactions.

Adverse reaction	ICD (*n* = 57) *N* (%)		CD (*n* = 53) *N* (%)	
	Any grade	Grade ≥3	Any grade	Grade ≥3
Anaemia	44 (57.2)	17 (29.8)	46 (86.8)	12 (22.6)
Thrombocytopenia	48 (84.2)	26 (45.6)	35 (66.0)	13 (24.5)
Neutropenia	30 (52.6)	17 (29.8)	28 (52.8)	13 (24.5)
Diarrhoea	31 (54.4)	6 (10.5)	29 (54.7)	1 (1.9)
Constipation	21 (36.8)	0	12 (22.6)	0
Nausea	26 (45.6)	2 (3.5)	22 (41.5)	0
Vomiting	15 (26.3)	2 (3.5)	13 (24.5)	1 (1.9)
Sensory neuropathy	18 (31.6)	3 (5.3)	9 (17.0)	0
Peripheral neuropathy	25 (43.9)	1 (1.8)	17 (32.1)	0
Fatigue	41 (71.9)	4 (7.0)	34 (64.2)	1 (1.9)
Infection	29 (50.9)	12 (21.1)	25 (47.2)	7 (13.2)
Thromboembolic event	0	0	1 (1.9)	0
Oedema/ankle swelling	18 (31.6)	0	10 (18.9)	0
Dyspepsia	7 (12.3)	0	2 (3.8)	0
Rash	12 (21.1)	0	7 (13.2)	0
Dehydration	5 (8.8)	0	3 (5.7)	0
Hypotension	2 (3.5)	0	9 (17.0)	2 (3.8)
Muscle weakness	7 (12.3)	2 (3.5)	8 (15.1)	1 (1.9)
Hyperglycaemia	1 (1.8)	0	8 (15.1)	2 (3.8)
Bleeding	8 (14.0)	3 (5.3)	5 (9.4)	1 (1.9)
Other	48 (84.2)	23 (40.4)	43 (81.1)	17 (32.1)

Sixty-five serious adverse events (SAEs) were experienced in 34 (59.6%) ICD participants, compared to 51 SAEs in 26 CD (49.1%) participants. Three serious adverse reactions (SARs) in the ICD arm resulted in death (pneumonia, intracranial haemorrhage and pleural empyema). No SARs resulted in death in the CD arm.

### Quality of life

Quality of life (QoL) questionnaire compliance at baseline was high at 97.3%, however this reduced at 3 and 6 months post-randomisation to 69.0% and 63.3%, respectively in the ICD arm, and 75.6% and 50.0%, respectively in the CD arm. Global health status was similar between the arms at baseline and 3 months, and slightly increased in the CD arm at 6 months (mean (SD) ICD: 59.3 (22.5), CD:67.2 (21.0)), although numbers are small (ICD:19, CD:15).

### ICD crossover

Twenty participants received crossover ICD treatment upon disease progression with CD. Median progression-free survival from day 1 cycle 1 of crossover treatment was 4.6 months (80% CI 4.1–5.0), with 17/20 participants progressing by the time of final analysis. 5/20 participants (25.0%) achieved at least a PR, including three VGPRs, with 10/20 (50.0%) participants achieving stable disease as their maximum response.

9/20 participants experienced a dose reduction of at least one treatment and 13/20 a dose omission (all including an ixazomib omission), with mean dose of ixazomib received 3.4 mg (SD 0.82). Thirty-one SAEs were reported in 15/20 participants, predominantly infections and infestations. Grade ≥3 adverse events reported in ≥20% patients were anaemia (25%), thrombocytopenia (55%), neutropenia (35%), and infection (20%).

## Discussion

MM therapy has undergone major changes in recent years, and outcomes have improved substantially in both the newly diagnosed and relapsed setting due to the widespread use of agents with novel mechanism of action. A true plethora of drug combinations have been studied in clinical trials, and many have been approved and are used in clinical practice [32]. Despite the undeniable clinical benefits of regimens combining novel agents [1, 2], many are costly or require frequent attendance at treatment centres. Moreover, novel triple or quadruple drug combinations are commonly evaluated in newly diagnosed patients or those with 1–3 prior lines of therapy, with trial entry criteria that are often selective for patients who are younger and fitter than many ‘real-world’ MM patients with advanced disease. Here, we show that the all-oral and comparatively inexpensive combination of ixazomib with cyclophosphamide and dexamethasone (ICD) did not improve key efficacy outcomes compared to cyclosphamide plus dexamethasone (CD), prior to treatment crossover in the CD arm. A likely explanations for why ICD was not superior to CD may be that the combination was too toxic for the patient population included. Patients enroled in MUKeight were not only comparatively old and heavily pre-treated, but were also frail. Based on the recently published simplified frailty score [31], 73% of patients enroled in MUKeight were classified as frail, and this number was even higher in the ICD arm (80.7%). This is substantially higher than the proportion of frail patients included in the BOSTON study (30%), which included a patients with 1–3 prior lines of therapy [33] and is one of the few randomised controlled trials so far to apply the simplified frailty score to post-hoc subgroup analyses [34]. However, the higher proportion of frail patients in MUKeight is naturally closer to the real-world situation outside of clinical trials, particularly in patients with advanced RRMM [35]. The notion that toxicity was a limiting factor for patients in the ICD arm of MUKeight is supported by the higher proportion of patients experiencing an SAE (59% vs. 49%), the higher number of ≥G3 AEs, and the greater proportion of ICD patients in whom doses were omitted. However, only marginally more patients in the ICD arm stopped treatment purely due to toxicity reasons (13.5% vs. 10.0%). In a small study of the all-oral triplet regimen of ixazomib with selinexor and dexamethasone, frequent treatment delays and dose reductions were also considered to be linked to a comparatively low response rate (ORR 22%) in patients with a median of 5 prior lines of therapy [36]. Given that ixazomib is widely reported to have an advantageous AE profile compared to bortezomib or carfilzomib [37], it appears unlikely that toxicity issues are specifically linked to ixazomib. Rather, our observations suggest that novel agent-containing triplet regimens should be evaluated very cautiously in frail or old patients, or those with very advanced disease. Such a notion may be supported by the favourable outcomes of ICD in NDMM patients, in whom a CR + VGPR rate of 33% and a PFS of 23.5 months was observed [19], while ICD in RRMM patients with 1–3 prior lines of therapy resulted in a ≥VGPR rate of 16% and a median PFS of 14.2 months [21]. The failure of ixazomib to improve outcomes could also be linked to reduced anti-MM activity in patients that had been exposed to a PI. However, the combination of ixazomib with pomalidomide and dexamethasone in bortezomib-resistant MM patients showed encouraging clinical activity [38], and the addition of ixazomib to lenalidomide and dexamethasone was associated with significantly longer PFS in PI exposed patients [13]. The optimal use of ixazomib in combination with cyclophosphamide and dexamethasone therefore remains to be established.

Patients randomised to CD in MUKeight had a median PFS of 6.7 months, with a DoR of 10.8 months and an OS of 19.1 months. However, OS analyses did not adjust for treatment crossover in the CD arm. While our findings do not provide a definite explanation for the relatively positive results observed with CD, the option for a crossover to ICD upon progression may have been instrumental given that 51% of CD patients achieved ≥MR. While not statistically significant, there was a clear trend of inferior OS in the ICD arm. In a post-hoc subgroup analysis, OS differences between the ICD and CD arms for both frail and non-frail patients did not reach significance levels, but suggest a possible detrimental effect of ICD in frail patients. While the data do not allow definitive conclusions on reasons for OS differences, the higher number of SAEs, ≥G3 AEs, and dose omissions in ICD patients suggests that excess toxicity may have been a relevant factor.

While cross-study comparisons need to be interpreted with great caution, it appears relevant to contextualise the key outcomes of the MUKeight trial. In a recent real-world analysis of all-oral pomalidomide, cyclophosphamide, and dexamethasone in RRMM patients with a comparable median age of 71 years who had received a median of three prior lines of therapy, ORR was 39%, with a median PFS of 7.6 months and OS of 12.6 months [39]. Thus, in patients of a similar age but with fewer prior therapies, major outcomes were comparable to those in MUKeight.

The MUKeight trial closed early due to continued slow recruitment, recruiting less than half of the pre-planned sample size of 250, and just short of the revised sample size of 140. Although the study did not recruit to revised target, with the event rate observed (96 PFS events), there was still 76% power to detect a treatment effect, i.e., the lack of effect is not due to lack of power. One likely reason for below-target recruitment is the UK National Institute for Health and Care Excellence (NICE) approval of ixazomib in combination with lenalidomide and dexamethasone in 2018 (https://www.nice.org.uk/guidance/ta505). It may also be argued that recruitment was affected by the inclusion of a CD arm, albeit with a permitted crossover to ICD treatment upon confirmed disease progression. The choice of CD as the comparator arm was based on several considerations. For patients with RRMM who have relapsed after treatment with thalidomide, lenalidomide and a proteasome inhibitor, CD was a standard treatment option in the UK when the trial was designed and opened. Indeed, CD remains a valid regimen for patients with advanced stage disease, particularly if they are unfit. The results of this study show that, at least in the frail and advanced patient population enroled, the inexpensive and all-oral combination of CD can indeed be associated with satisfactory responses, a finding that is particularly relevant for MM patients who do not have access to costly novel drug combinations.

In summary, the addition of ixazomib to cyclophosphamide and dexamethasone did not improve outcomes in the comparatively frail, old, and heavily pre-treated RRMM patients enroled in the MUKeight trial.

## Supplementary information

Supplementary material
