# Supplementary material for: Ixazomib with cyclophosphamide and dexamethasone in relapsed or refractory myeloma: MUKeight phase II randomised controlled trial results
Source: Blood Cancer J. 2022 Apr 1;12(4):52. doi: 10.1038/s41408-022-00626-4 (PMC8972903; doi:10.1038/s41408-022-00626-4)
Supplement: Supplementary file 1 — Supplementary material [file 41408_2022_626_MOESM1_ESM.pdf]

Supplementary material

Table S1 Mean dose of study drug received

	Mean (SD)	
**Treatment drug**	**ICD (n=57)**	**CD (n=53)**
Dexamethasone (mg)	27.2 (12.42)	27.1 (11.99)
Cyclophosphamide (mg)	458.4 (96.45)	473.2 (68.62)
Ixazomib (mg)	3.7 (0.69)	N/A

Table S2 Reasons for treatment discontinuation

	ICD (n=52)	CD (n=50)	Total (n=102)
**Reason for stopping treatment**			
Disease progression	31 (59.6%)	33 (66.0%)	64 (62.7%)
Disease progression, Clinician decision	0 (0.0%)	1 (2.0%)	1 (1.0%)
Disease progression, Intolerability due to toxicity	1 (1.9%)	0 (0.0%)	1 (1.0%)
Disease progression, Withdrawn consent	1 (1.9%)	0 (0.0%)	1 (1.0%)
Intolerability due to toxicity	7 (13.5%)	5 (10.0%)	12 (11.8%)
Clinician decision	3 (5.8%)	4 (8.0%)	7 (6.9%)
Clinician decision, Other	2 (3.8%)	0 (0.0%)	2 (2.0%)
Died	3 (5.8%)	2 (4.0%)	5 (4.9%)
Withdrawn consent	1 (1.9%)	4 (8.0%)	5 (4.9%)
Withdrawn consent, Clinician decision	1 (1.9%)	0 (0.0%)	1 (1.0%)
Other	2 (3.8%)	1 (2.0%)	3 (2.9%)

Other reasons: ICD: clinician decision, ECOG3 (x2); patient and clinician decision; clinician decision, Cycle 10 was delayed due to suspected progression which was excluded. Further delay was caused by a suspicious endometrial malignancy, which was again excluded; CD: pt not received previous thal,

Figure S1 Dose modification schedule

	Dose Reductions		
	**Ixazomib**	**Cyclophosphamide**	**Dexamethasone**
**Starting dose**	Ixazomib 4 mg<br>Days 1, 8 and 15	Cyclophosphamide 500 mg<br>Days 1, 8 and 15	Dexamethasone 40 mg PO Days 1-4 and 12-15
**Starting dose for older/less fit participants**			Dexamethasone 20 mg PO<br>Days 1-4 and 12-15
**1st dose reduction**	Ixazomib 3 mg<br>Days 1, 8 and 15	Cyclophosphamide 400 mg<br>Days 1, 8 and 15	Dexamethasone 20 mg PO<br>Days 1-4 and 12-15
**2nd dose reduction**	Ixazomib 2.3 mg<br>Days 1, 8 and 15	Cyclophosphamide 300 mg<br>Days 1, 8 and 15	Dexamethasone 10 mg PO<br>Days 1-4 and 12-15
**3rd dose reduction**	Ixazomib Discontinue	Cyclophosphamide 200 mg<br>Days 1, 8 and 15	Dexamethasone 10mg PO<br>Days 1-2 and 12-13

Please Note:

- ***Doses should not be re-escalated.***
- ***If dexamethasone has to be discontinued, cyclophosphamide and ixazomib must also be discontinued.***
- ***If ixazomib is discontinued participants may continue with cyclophosphamide and dexamethasone alone***
